# The virtues of limits and environmental sustainability in healthcare

**DOI:** 10.1111/bioe.13382

**Published:** 2025-01-22

**Authors:** Xavier Symons

**Affiliations:** ^1^ Plunkett Centre for Ethics Australian Catholic University Darlinghurst New South Wales Australia

**Keywords:** climate, energy policy, environment, resource allocation, virtue ethics

## Abstract

The spectre of human‐induced climate change has drawn attention to the need to discover new, environmentally sustainable approaches to healthcare. This article draws upon David McPherson's *The Virtues of Limits* (2021) to develop a virtue ethics for sustainability in healthcare. I explore how a virtuous appreciation of the value of healthcare resources can lead us to exercise stewardship in our use of those resources, whereas a failure to appreciate the value of resources can lead to irresponsible and unsustainable patterns of use. I close with some recommendations of how health systems might go about facilitating the cultivation of the virtues of sustainable health practice.

## 1 INTRODUCTION

The global community has responded to the spectre of human‐induced climate change by seeking more sustainable forms of environmental resource use and altering energy production and consumption. One area of focus has been health systems, which are invariably one of the biggest energy consumers and waste producers in an economy. But the immediate and direct goal of saving lives and restoring people to health in a health system may conflict with long‐term and indirect attempts to reduce the severity of climate change and environmental degradation by altering the way in which health systems utilize energy and other environmental resources. This presents a problem for the sustainability project and necessarily forms the backdrop of any realistic conversation about environmental sustainability in healthcare. We might think of this problem as intersecting with the widely discussed *rule of rescue* problem in bioethics.^1^ On the one hand, you have identifiable lives in the health system at present that need saving and restoration to health, while, on the other hand, you have a broader future problem of environmental depletion and resource scarcity as a result of the unsustainable consumption of resources in the present. How ought we respond to this dilemma?

This paper explores the ethics of sustainable resource use in health systems through the lens of neo‐Aristotelian virtue ethics and argues for the local, micro‐level cultivation of what scholars call *the virtues of limits*,^2^ or appropriate responsiveness to the limits that human value places on human action, and *the virtues of sustainability*, or virtues that are constitutive of sustainable modes of human living.^3^ This paper will posit a link between how individual actors in the health system might approach more traditional resource allocation dilemmas and more contemporary, environmentally focused questions concerning the use of resources by the health system. A strong recognition of our creaturely status within nature–a perspective that is consonant with neo‐Aristotelian ethical naturalism and Stoic thought—will be reflected in a certain circumspection with which we approach the use of resources, be they energy resources or discrete medical interventions. The virtues that one ought to practice in the context of environmental sustainability—such as acceptance, gratitude, humility, and justice—are the same virtues that one ought to practice in the context of healthcare rationing more conventionally understood. As such, a comprehensive virtue ethical framework emerges for ethical resource use in the health sector which applies both to medical interventions as well as energy and equipment utilized.

## 2 THE CHALLENGE OF SUSTAINABILITY IN HEALTH SYSTEMS

Environmental sustainability is a principle that is invoked in the context of discussions about economic and social development that describes a pattern of growth and consumption that does not damage or deplete the environment in such a way that future generations are prevented from accessing sufficient resources to meet their needs. That is to say, environmental sustainability has as its proper object the prudent and non‐exhaustive use of environmental resources over time. Another term in the literature which is basically equivalent is *sustainable development*.^4^

Global efforts to achieve environmental sustainability have gained momentum in recent years as concerns about climate change, biodiversity loss, pollution, and resource depletion have risen. The United Nations’ 2030 Agenda for Sustainable Development, for example, includes 17 sustainable development goals (SDGs) that address a range of global challenges, including environmental sustainability. Goals such as clean water and sanitation, affordable and clean energy, climate action, life below water, and life on land emphasize the interconnectedness of social, economic, and environmental challenges.^5^ Similarly, The Paris Agreement, adopted at the 21st Conference of the Parties (COP21) in 2015, is an international accord that aims to limit global warming to well below 2°C above pre‐industrial levels. Countries commit to nationally determined contributions (NDCs) to reduce greenhouse gas emissions and enhance their resilience to the impacts of climate change.^6^

But change at the level of entire economies presupposes major change in individual economic sectors such as the health sector. Health systems are one of the biggest consumers of energy and their problematic patterns of consumption have recently come into focus. In the United States, for example, the health sector accounts for 8.5% of carbon emissions.^7^ The industry would rank 13th in the world in terms of greenhouse gas output if it were a country.^8^ In addition to extensive energy consumption, health systems also consume large volumes of waste, both plastics and textiles as well as medical waste, and much of this waste is difficult to recycle or even dispose of in a manner that does not damage the environment. The U.K. data, for example, indicates that “on average, the NHS currently produces an estimated 156,000 tonnes of clinical waste every year from secondary care,” and that “[t]he estimated carbon impact of this waste is approximately 100,000 tonnes of CO2e per year.”^9^ Estimates suggest that “[h]ospital patients in the United States generate about 33.8 pounds of waste each day, which leads to about 6 million tons of waste annually.”^10^

Some health systems are making serious efforts to reduce their carbon footprint and waste output. In 2022, for example, the National Health Service became the first health service to embed a net zero emissions goal into legislation, pledging to achieve this target by 2040. This legislative commitment was accompanied by an extensive report that outlined an iterative and adaptive approach for achieving this target. This report is considered to be statutory guidance and is functioning as a blueprint for the development of specific policies.^11^

But the challenge of balancing the immediate health needs of a population with long‐term goals of sustainability remains to be seen. This situation is exacerbated by various economic and social pressures. It is difficult, for example, to market eco‐friendly boilers as an alternative to gas boilers in hospitals which already have tight capital works budgets and may be recovering from periodic resource strains such as the COVID‐19 pandemic. Alternatively, political opposition to climate action in some jurisdictions makes meaningful change difficult or, in some cases, virtually impossible.^12^ In the United States, for example, there is a wide gulf among Democrat and Republican voters over whether climate change ought to be a top priority for the President and Congress, with Republican voters being significantly less likely to hold this opinion.^13^

Granted, healthcare organizations have made significant improvements in reducing their environmental footprint, including efforts to identify climate‐related risks and opportunities over the short, medium, and long term to inform strategic planning and development of resource transition plans. Healthcare organizations have become more transparent through detailed sustainability reporting to key stakeholders focusing on sustainability goals. Carbon reduction initiatives such as green energy procurement and waste management consolidation and optimization initiatives are becoming increasingly common. Waste diversion exercises (diverting waste from landfill through filling council recycling and green disposal bins with appropriate waste) or plastic‐free time periods in an organization (e.g., “plastic‐free July”) are examples of such initiatives.

But tensions still remain about which approach is the most appropriate. Given these tensions, are there ethically informed approaches to addressing the environmental challenges of our time that do not conflict with the economic and moral imperative of hospitals to meet current health needs? The remainder of this paper will provide an overview of virtue ethics and, specifically, the virtues of limits and sustainability that constitute an ethical response to the environmental harms that stem from unsustainable health practices. This approach may avoid some of the political and economic complexities surrounding climate action today, while still speaking to the need for sustained change in patterns of use of interventions, equipment, and energy.

## 3 VIRTUE ETHICS

Virtue ethics is an approach to ethics that treats character as morally basic and focuses on excellence of character or virtues. In contrast to other ethical systems, the defining question for virtue ethics is not “how ought I act?” but rather “how ought I live?” or “who ought I become?” That is to say, virtue ethics is focused on general patterns of behavior or modes of life more than acting correctly in particular moral situations. It is an aretaic ethical framework (ἀρετή (arete), meaning excellence) as opposed to deontic ethical frameworks (δέον (deon), meaning duty).

Virtue ethics has its origins in Ancient Greek moral philosophy, and, specifically, Aristotle's ethical writings, though it should be stressed that not all variants of virtue ethics are Aristotelian. Aristotle's agent‐relative ethics can be contrasted with modern ethical systems such as utilitarianism or deontology which arise from an impartialist approach to ethics articulated in the work of late Enlightenment thinkers such as Immanuel Kant or Jeremy Bentham and John Stuart Mill.^14^ The deontological system of Kant is firmly focused on the right‐making features of human action that are generalizable to all rational agents while the utility‐maximizing systems of Bentham and Mill are focused on an objective assessment of the consequences of action. These systems are only secondarily if at all focused on moral character. For Aristotle, in contrast, moral character and the human good were of fundamental concern and are prior to an articulation of one's moral obligations.

Importantly, virtues are not superficial aspects of personality or personal taste but rather deep features of human psychology and moral rationality. According to Aristotle, virtues lead one to respond to the right thing for the right reason and in the right way. But this requires an orientation of one's whole being toward virtue. Thus, we can say that:

A virtue is an excellent trait of character. It is a disposition, well entrenched in its possessor—something that, as we say, goes all the way down, unlike a habit such as being a tea‐drinker—to notice, expect, value, feel, desire, choose, act, and react in certain characteristic ways. To possess a virtue is to be a certain sort of person with a certain complex mindset.^15^

Virtues are stable patterns of behavior, deeply seated in the faculties of a human being that lead someone to act in consistent ways over time and across a range of situations. For example, it would not suffice to be the possessor of a virtue like honesty and to only tell the truth in a certain subset of situations. One must be consistently honest in a way that does not vary with circumstance or interlocutor.

It is also important to emphasize the close connection in Aristotle's writings between the flourishing of an individual person and their situatedness in society (specifically, the city‐state). Aristotle writes that “man is a political animal (ζῷον πoλιτικόν)” (1253a1).^16^ The *Nicomachean Ethics* is framed as a preface for the *Politics*, Aristotle's seminal work on political philosophy and the nature and proper function of the city‐state. Aristotle was of the view that the “end (τέλος) of individuals and of states is the same” (1334a13) or, alternatively, that the “happiness (εὐδαιμονία) of the individual is the same as that of the state” (1324a5‐6).^17^ The cultivation of both moral and intellectual virtue is a thoroughly social process, and the state, according to Aristotle, plays an important role in the moral cultivation of its citizens from youth until adulthood.

Virtue ethics has undergone a major revival in recent decades. Initially, Neo‐Aristotelian virtue ethics was proposed as a more grounded alternative to the abstract and impersonal philosophical systems of the high‐modern era as well as a way to address the problem of moral terminology like “obligation” that, in the modern era, had become unmoored from its classical or theistic philosophical context,^18^ More recently, virtue ethics has been widely explored in the context of positive psychology, character and virtue studies in educational theory (Kristjansson 2016), and professional ethics.^19^

## 4 THE INTERSECTION OF VIRTUE ETHICS AND ENVIRONMENTAL SUSTAINABILITY

Someone might, however, wonder what advantages arise from bringing the lens of Aristotelian virtue ethics to bear on the problem of environmental sustainability. This section offers some brief considerations as an initial response to this question.

Most importantly, virtue ethics fills an important gap in ethical approaches to sustainability, namely, the need to perfect the moral character of individual agents in addition to affecting macro‐level policy reform. That is to say, what is needed for sustainability is not just good theory and policy frameworks but also stable character traits that will ensure that people act in an environmentally sustainable way across a variety of situations and across an extended period of time. Virtue ethics tracks this consideration. Environmental virtue ethics focuses on “sustained commitment, the development of communities of agents, and the importance of doing one's part even when others fail to do theirs.”^20^

Sustainability, after all, has as its ultimate point of reference the flourishing of human communities across generations, and, as such, ought to focus on fostering patterns of behavior and dispositions of character that allow for sustained positive change. Longtermist philosophers like William MacAskill have attempted to make the case for a change in moral theory that avoids presentist bias and that recognizes that “[people] matter even if they live thousands of years hence” (p. 10). Masckill writes in *What We Owe to the Future* of the phenomenon of value lock‐in, or “an event that causes a single value system, or set of value systems, to persist for an extremely long time” (p.78). For MacAskill, we ought to maintain some sort of plasticity to our values or at least recognize the importance of achieving *value lock‐in* of the right kinds of values–values that will be conducive to the long‐term survival and flourishing of humanity.

Sustained change, however, is not only about a change of thinking where we include future persons in our moral calculation nor is it limited to structural features of societies that promote good action; it is also about a change of heart at the level of individual moral agents. Human moral frailty is at the heart of many of the kinds of existential risks that longtermists tend to highlight as posing the greatest risk to humanity—nuclear war or climate change—with the exception of, perhaps, artificial intelligence and a deadly pandemic. Addressing human frailty, then, is a central part of bringing about lasting change. Virtue ethics speaks to this task by focusing on the character and psychology of individual moral exemplars who embody ideals like justice and temperance.

Environmental policy discourse can, of course, focus on utility or justice in discussions of sustainability. This has been the case with effective altruist arguments for investment in green energy solutions or the discourse surrounding “climate justice.” Indeed, there may be value in appealing to benefits or obligations when structuring macro‐level policy discussions about the environment. But we cannot in the process lose track of the centrality of moral character in achieving lasting change. In addition, the rhetoric of movements like the climate justice movement can come across as doctrinaire and ideologically problematic and less loaded virtue language may be more palatable and have wider appeal.

At a deeper level, one could argue that Aristotelian virtue ethics is uniquely well‐suited for conceptualizing environmental sustainability because it is focused on behaviors and ways of living that are fundamental to human development but that arguably also presuppose a recognition and acceptance of the proper place of the human person within nature. Aristotle's philosophical vision was, after all, predicated on an analysis of embodied human nature and the proper function of a human being within the natural environment. Building on this approach, Neo‐Aristotelian philosophy has developed an account of the human good that resists the philosophical abstraction and instead seeks to ground the human telos in the natural history of human beings (Foot, 2003). It seems fitting that this non‐reductive philosophical naturalist framework be extended to an account of the natural world that reflects an understanding of our creaturely position within a cosmos and presupposes an awe and respect for nature as both resource and gift. Rather than viewing nature in opposition to the assertion of human will and human mastery, we can instead view nature as the natural home of the human person and supportive of the realization of our natural ends. Perhaps one might argue that these ideas are even more at home in the philosophical worldview of Stoic philosophers like Epictetus or Marcus Aurelius. But Stoic philosophers like Epictetus arguably gave less attention to the positive side of emotions and their capacity to orient virtuous individuals toward the good. A Stoic worldview lends itself less to the idea of orienting oneself toward sustainability at all levels of one's being including emotions.

## 5 THE VIRTUES OF LIMITS AND THE VIRTUES OF SUSTAINABILITY

But it is necessary to go deeper than these insights, as questions arise about what kinds of virtues are most important in embodying an ethic of awe and respect for the natural environment. The following section will attempt to synthesize insights in recent virtue ethics scholarship, with a focus on David McPherson's 2021 book *The Virtues of Limits* and a recent volume entitled *The Virtues of Sustainability* edited by Jason Kawall.^21^ This will provide a further articulation of how precisely virtue ethics intersects with environmental sustainability.

*The Virtues of Limits* offers a defense of the place of limits in human life and, moreover, articulates a list of virtues that constitute a proper responsiveness to basic human values or goods. McPherson synthesizes a long thread of philosophical reflection–running through Plato and Aristotle through GK Chesterton and Philippa Foot to GA Cohen and Michael Sandel–to capture what it means to live a life cognizant of the embodied, finite, and, therefore, limited character of human existence. McPherson's insights can usefully scaffold our understanding of what it means for agents in different sectors of human society to use resources in a sustainable manner.

Central to McPherson's argument is a distinction between two fundamental existential stances that one might adopt toward human nature and the world, namely, the *choosing and controlling stance* and the *accepting and appreciating stance*. The choosing and controlling stance refers to an approach to the human condition whereby one seeks to take radical control over human limits and to transcend the finite, fragile, and fallible parameters that characterize the zone of human action. We might think of this as a Promethean desire to overcome the constraints that human nature impose on us and our freedom. Indeed, this is particularly relevant in the health sphere, where biotechnologies are increasingly allowing us to alter fundamental aspects of human physiology that might previously have been unimaginable. In an essay from 2000 entitled “Playing God: Genes, Clones, and Luck,” Ronald Dworkin suggested that, rather than being categorically opposed to “playing God” in the context of genetic engineering, we should, in fact, make a virtue of launching out into the unknown and boldly taking control of our biological destinies:

Playing God is indeed playing with fire. But that is what we mortals have done since Prometheus, the patron saint of dangerous discovery. We play with fire and take the consequences, because the alternative is cowardice in the face of the unknown. (Dworkin 2000, p. 446)

He goes on to note that:

[If] playing God means struggling to improve our species, bringing into our conscious designs a resolution to improve what God deliberately or nature blindly has evolved over eons, then the first principle of ethical individualism commands that struggle, and its second principle forbids, in the absence of positive evidence of danger, hobbling the scientists and doctors who volunteer to lead it. (Dworkin, 2000, p. 452).

Dworkin is not the only bioethicist to suggest that we have an imperative to use science to improve human wellbeing even when this crosses the line between therapy and enhancement. It is, rather, a relatively common commitment in academic bioethics today.

The accepting and appreciating stance, in contrast, refers to an approach to human life that accepts and takes as normative the fundamental limits that characterize our nature and that seeks to live and thrive within these limits. It is not that the accepting and appreciating stance is quietistic and refuses to seek to improve human wellbeing by overcoming sickness, injury, and disease. We *should* struggle to fight disease just as we should seek to improve the material and economic circumstances of humankind. McPherson's point is, rather, that we should always pursue these aims with a healthy and realistic sense of our limits as human beings and, indeed, cognizant of the role that these limits play in structuring and making available certain basic goods for our choosing. An accepting and appreciating stance, for example, might aspire to lengthen human life through certain medical interventions but not to extend life indefinitely as if immortality were a final goal of medicine.

What, specifically, are these limiting virtues? McPherson focuses on six virtues that are characteristic of the accepting and appreciating stance. These are, namely, reverence, contentment, humility, neighborliness, loyalty, and moderation. There is much that could be said about each of these virtues but suffice to say that they each are important structuring features of the accepting appreciating stance and its implications for how we approach the existential, moral, social, and economic limits that characterize our lives. McPherson takes humility to be a kind of master virtue that underpins a proper orientation toward the world. Thus, he writes:

[Humility] ensures we recognize and live out our proper place in the scheme of things, and it is concerned, among other things, with reining in the tendency to “play God.” (McPherson 2021, p. 29).

Humility is the counterpoint to the virtues of hubris that Dworkin and Nietzsche before him have defended. Rather than seeking to overcome all moral and metaphysical constraints on action, we instead aspire to appreciate and grow within the parameters that morality and nature bequeath to our human existence.

Living out our proper place within the scheme of things has implications for how human beings situate themselves and act within the natural environment. An accepting and appreciating stance is one in which we accept and appreciate our dependence on the natural environment from which we utilize resources and that seeks to use these resources in a manner cognizant of that dependence and of the gifted character of natural resources. In contrast, a choosing and controlling stance would view the natural world through a lens of domination and control and might instead seek to exercise technological mastery over the world that fundamentally depletes the natural environment and disrupts natural patterns of growth, development, and replenishment of natural resources. The philosophy behind the virtues of limits thus provides a check on unbridled patterns of consumption in healthcare and other sectors of the economy. It leads to a framework where we embrace our dependence on the natural environment in a consistent manner whereby our patterns of consumption are cognizant of the needs of future generations.

Echoes of this paradigm are found in recent literature on *the virtues of sustainability*. In his 2021 edited volume, Kawall describes the virtues of sustainability as “those virtues that will play an especially important role in allowing us to pursue and lead sustainable, flourishing lives” (p. xxvi). While all the virtues will to some extent be relevant to this goal, there are, however, certain virtues that are of particular importance. In his summary of the scholarship, Kawall mentions in particular humility (echoing McPherson) and simplicity. On the topic of humility, Kawall notes:

…humility can help us to overcome hubris with respect to the human ability to control or dominate nature, or problematic anthropocentric assumptions about what has intrinsic value; it can help us to see ourselves as truly embedded in, and dependent upon, the natural systems of this planet. (p. xxvi).

Similarly, on simplicity, he reflects that:

A virtue of simplicity would encourage reflective, thoughtful decisions with respect to consumption—what sorts of goods do we really need? Can we find better, less consumption‐driven ways of life? (p. xxvi).

Contributors to Kawall's edited volume focus also on patience, cooperation, conscientiousness, creativity, and open‐mindedness. At risk of oversimplification, these virtues all aim at long‐term change and consider what it would take to affect meaningful collective action on climate change. Paradoxically, what is required is a careful but also creative and open approach to considering how we each individually might change our patterns of consumption and resource use. The virtues of sustainability are imaginative virtues whereby we attend more closely to resource use and fix our attention on reimagining how societies may be structured and operate in a more sustainable manner, starting with our own personal behavior.

One final comment on the broad political appeal of the virtues of sustainability is order. In essence, there are political reasons to adopt a virtuous ethical approach to sustainability given that it is predicated on an acceptance of certain objective, metaphysical, ordering principles to the universe (κόσμος (cosmos)) and our position as creatures within that ordered universe. This approach may resonate with stakeholders with more conservative political leanings insofar as it tracks certain religious and realist metaphysical intuitions about the objective, mind‐independent structure of the world while at the same time highlighting the implications of these intuitions for the way we relate to the natural environment. As such, it may present a way to transcend political polarization of the issue of environmental sustainability and provide at least one area of consensus on how we might think about societal challenges in this area.

## 6 RELEVANCE TO HEALTHCARE

Healthcare seeks to halt the progress of disease, injury, and even aging. This is a noble aspiration and is reflective of a fundamental desire to improve human wellbeing. But there are limits on what we can reasonably be expected to achieve as limited beings caring for limited beings. This insight ought to underpin any discussion of the allocation of scarce healthcare resources. But it also applies in the context of sustainable patterns of living and an appreciation for the resources that we do have at our disposal. That healthcare is possible is itself something that we can be grateful for and, conversely, some degree of resource scarcity is something that we must accept.

Do the virtues of limits and sustainability provide us with guidance for how we ought to approach trade‐offs between saving lives and promoting health, on the one hand, and caring for the environment, on the other? Recent scholarship suggests that individual character matters and that we need, as a minimum, to think about our individual patterns of consumption even if we may, at a macro‐level, be limited in our ability to limit use of the environment to provide for the needs of the health system. Indeed, part of existential humility is accepting our limited ability to control what occurs at the highest levels of collective action. But virtues like creativity also do lead us to think imaginatively about how we can make health systems more sustainable.

These virtues lead us to challenge the supposed fixed constants of health systems and to think whether indeed there is a different way of operating health systems—one that is at once more sustainable but also still observant of the therapeutic imperative of healthcare. Thus, we can say that there is an overlap between virtues that might prompt restraint in the use of aggressive healthcare interventions and the virtues that might lead one to use energy resources more sustainably. Someone who is inordinately concerned with efficiency and health outcomes might administer aggressive health interventions in an inappropriate manner while also neglecting to carry out basic steps that he or she, as an individual actor in the health system, might undertake to reduce environmental impact. This would be an example of hubris, insofar as it exhibits a wantonness with respect to nature both, as it manifests in the body of a patient as well as in the resources that we utilize to provide healthcare.

In contrast, a doctor might reflect on how he or she can reduce a patient's length of stay where appropriate and avoid duplicate or unnecessary tests or procedures. Among other things, this would be an example of existential humility, insofar as the doctor has an appreciation of the limits of healthcare and the need to use resources in the health system in a manner that reflects the finite nature of resources as such and also the burden that excessive interventions place on a patient's quality of life.

Perhaps these remarks may appear too general to meet the critique that virtue ethics fails to yield concrete guidance for action.^22^ In response, we might consider some additional practical guidance yielded by the virtues of limits and the virtues of sustainability. We might consider how the virtues of sustainability apply to appropriately recycling used medical products. According to the WHO, approximately 85% of healthcare waste is similar to domestic waste, so it can be recycled.^23^ Some forms of medical waste need to be stored for many years, such as radioactive waste, and so perhaps the issue here is more of avoiding unnecessary use in the first place. Latex, PVC, and mercury have been flagged as hazardous materials of which the health sector could reduce use.^24^ Anesthetics are also in some cases harmful to the environment; in some cases, alternatives can be utilized.^25^

Part of limiting medical waste will involve structural solutions that concern whole health systems. But presumably, it will also require individual clinicians to exercise prudence as to the resources that they themselves utilize in their clinician practice. Individual clinicians, furthermore, can be judicious in the kinds of medical equipment and drug suppliers that they utilize for their work. Certain medical device manufacturers are committed to sustainable medical device manufacturing while some drug companies are consciously making changes to production and delivery to reduce their environmental impact. Thus, a virtuous ethical approach to sustainability would presumably be a useful complement to higher level structural solutions to excess consumption of medical resources.

But we also ought to return to the fundamental premise of virtue ethics, namely, that virtue and character are intrinsically valuable and that, even if the net effect of the cultivation of the virtues of limits on carbon emissions or energy consumption was limited, it would still be worthwhile to encourage the cultivation of these virtues. Sustainable communities, after all, are not just characterized by systems of resource use that are viable for the future but also people who have a mentality that recognizes the limits of human life and our responsibilities to future generations. This mentality will not only be achieved at global level but also through the cultivation of individual character. Indeed, Pope Francis has written of the value of personal changes in effecting lasting personal change. In *Laudate Deum*, he writes that:

The mere fact that personal, family and community habits are changing is contributing to greater concern about the unfulfilled responsibilities of the political sectors and indignation at the lack of interest shown by the powerful. Let us realize, then, that even though this does not immediately produce a notable effect from the quantitative standpoint, we are helping to bring about large processes of transformation rising from deep within society.^26^

Ultimately, it is these “large processes of transformation rising deep within society” that the virtue ethical approach to sustainability is primarily concerned with. The proper object of a virtue ethics of sustainability is the human person and their posture toward the world, and, by extension, the view of the environment reflected in the way that our societies are organized. Change will radiate out from individual moral actors toward the broader community.

It would be amiss not to mention the role of reflection and attention in reorienting health systems to sustainability. In addition to any initiatives that health organizations institute to help make their institutions more sustainable, they must also create spaces for staff to reflect on the way in which they interact with the natural environment. This could take the form of regular workshops with board and executive of healthcare organizations, as well as short retreats or reading groups for clinical staff where opportunities are created to reflect on how one is comporting oneself with respect to the environment as well as the resources at one's disposal. These sorts of initiatives are practically non‐existent in the healthcare sector and this may represent a promising direction for future practice innovation and evaluation. The literature on mindfulness and sustainability could be a useful guide for pilot initiatives in this space.^27^

## 7 CONCLUSION

This essay has focused on the virtues of limits and sustainability and has sought to show how these virtues are an important dimension of achieving environmental sustainability within health systems. Importantly, these virtues are underpinned by a fairly radical change of heart. It seems that something more than just the policy case for the climate crisis is needed. Attentiveness to the world is needed. Deep change is needed. And this is what a recognition of human limits can yield.

The virtues of limits and sustainability should not be conceived of as an either/or in the context of sustainability policy in health systems but rather a both/and, and perhaps even a first step that can be proposed notwithstanding higher level debates about the economic cost of sustainability and the immediate responsibilities of health systems. The focus of the virtues of limits and sustainability is on character, rather, and fundamental perspective rather than the kind of technology we employ. It would affect the way we utilize resources, but not in a manner that would anyway undermine a holistic pursuit of the goals of healthcare viewed within the context of the broader flourishing of individuals and societies.

Thus, the virtue ethics articulated in this essay is not fundamentally individualistic. On the contrary, we ought to keep in view the communitarian dimension of virtue according to Aristotle and the role of communities in educating people in virtue. Health systems need to allow healthcare practitioners space to establish more sustainable health practices at a local level. But paradoxically, this will involve creating a broader culture in which such change is welcomed and indeed encouraged. Reform at a macro‐level and reform at a micro‐level are mutually reinforcing. The primary way in which health systems might achieve change, then, is by complementing broader structural change with a less systems‐based approach to sustainability and introducing messaging that focuses on what each individual actor within health systems can do, notwithstanding constraints on agency that modern health bureaucracies present. Creative and responsible agency is, after all, central to an ethic of sustainability.

## Footnotes

^1^
Jonsen, A. R. (1986). Bentham in a box: Technology assessment and health care allocation. *Law, Medicine and Health Care*, *14*, 172–174; McKie, J., & Richardson, J. (2003). The rule of rescue. *Social Science and Medicine*, *56*(12), 2407–2419. https://doi.org/10.1016/s0277-9536(02)00244-7

^2^
McPherson, D. (2021). *The virtues of limits*. Oxford University Press.

^3^
Kawall, J. (Ed.). (2021). *The virtues of sustainability*. Oxford University Press.

^4^
Brundtland, G. H. (1987) *Our common future: Report of the World Commission on Environment and Development* (UN‐Dokument A/42/427).

^5^
UN DESA. (2023). *The Sustainable Development Goals Report 2023: Special Edition—July 2023*.

^6^
United Nations Framework Convention on Climate Change. (2018). *The Paris Agreement*. United Nations.

^7^
Seervai, S., Gustafsson, L., & Abrams, M. K. (2022, April). How the U.S. health care system contributes to climate change. The Commonwealth Fund. https://doi.org/10.26099/m2nn-gh13.

^8^
American Medical Association. (2022). *Fight climate change and save money—Go green at your practice*. American Medical Association.

^9^
National Health Service England. (2023). *NHS Clinical Waste Strategy*. National Health Service England.

^10^
Navami, J., & LaBeaud, D. (2022). How should US health care lead global change in plastic waste disposal. *AMA Journal of Ethics*, *24*(10), E986–E993. https://doi.org/10.1001/amajethics.2022.986

^11^
National Health Service England. (2022). *Delivering a Net Zero National Health Service*.

^12^
Kamark, E. (2019). *The challenging politics of climate change*. The Brookings Institution. https://www.brookings.edu/articles/the-challenging-politics-of-climate-change/.

^13^
Kennedy, B., & Hefferon, M. (2019). U.S. concern about climate change is rising, but mainly among Democrats. Pew Research Center. https://www.pewresearch.org/fact-tank/2019/08/28/u-s-concern-about-climate-change-is-rising-but-mainly-among-democrats/

^14^
Newey, C. (2022). Impartiality in moral and political philosophy. *Oxford Research Encyclopedia of Politics*. Retrieved March 25, 2024, from https://oxfordre.com/politics/view/10.1093/acrefore/9780190228637.001.0001/acrefore-9780190228637-e-2015

^15^
Hursthouse, R., & Pettigrove, G. (2023). Virtue ethics. In E. N. Zalta & U. Nodelman (Eds.), *The Stanford Encyclopedia of Philosophy*. https://plato.stanford.edu/archives/fall2023/entries/ethics-virtue/

^16^
Aristotle, *Politics* 1.1253a.

^17^
Aristotle. *Nicomachean Ethics*.

^18^
Anscombe, E. (1958). Modern moral philosophy. *Philosophy*, *33*(124), 1–16; Baril, A., & Hazlett, A. (2019). The revival of virtue ethics. In I. Thomson & K. Becker (Eds.), *Cambridge history of philosophy: 1945–2015* (pp. 223–236). Cambridge University Press.

^19^
Seligman, M. (2010). *Flourish: A new understanding of happiness and wellbeing*. Nicholas Brearley Publishing; Kristjánsson, K. (2016). Flourishing as the aim of education: Towards an extended, ‘enchanted’ Aristotelian account. *Oxford Review of Education, 42*(6), 707–720. https://doi.org/10.1080/03054985.2016.1226791; Pellegrino, E., & Thomasma, D. (1993). *The virtues in medical practice*. Oxford University Press.

^20^
Sandler, R. (2013). Environmental virtue ethics. In L. F., Hugh (Ed.), *The Internet encyclopedia of ethics* (p. 7). Wiley.

^21^
McPherson, op. cit. note 2; Kawall, op. cit. note 3.

^22^
Begley, A. M. (2005). Practising virtue: A challenge to the view that a virtue centred approach to ethics lacks practical content. *Nursing Ethics*, *12*(6), 622–637. https://doi.org/10.1191/0969733005ne832oa

^23^
World Health Organization. (2018). *Health‐care waste*. https://www.who.int/news-room/fact-sheets/detail/health-care-waste

^24^
Jameton, A., & Pierce, J. (2001). Environment and health: 8. Sustainable health care and emerging ethical responsibilities. *CMAJ*, *164*(3), 365–369.

^25^
Yasny, J. S., & White, J. (2012). Environmental implications of anesthetic gases. *Anesthesia Progress*, *59*(4), 154–158. https://doi.org/10.2344/0003-3006-59.4.154; Varughese, S., & Ahmed, R. (2021). Environmental and occupational considerations of anesthesia: A narrative review and update. *Anesthesia & Analgesia*, *133*(4), 826–835. https://doi.org/10.1213/ANE.0000000000005504

^26^
Francis. (2023). *Laudate Deum*. Vatican Publishing House.

^27^
Ericson, T., Gunaketu Kjonstad, B., & Barstad, A. (2014). Mindfulness and sustainability. *Ecological Economics*, *104*, 73–79.

